# Plasma Trimethylamine-N-oxide following Cessation of L-carnitine Supplementation in Healthy Aged Women

**DOI:** 10.3390/nu11061322

**Published:** 2019-06-13

**Authors:** Joanna J. Samulak, Angelika K. Sawicka, Emilia Samborowska, Robert A. Olek

**Affiliations:** 1 Faculty of Rehabilitation and Kinesiology, Department of Bioenergetics and Nutrition, Gdansk University Physical Education and Sport, Gorskiego 1, 80-336 Gdansk, Poland; joanna.samulak@awf.gda.pl (J.J.S.); angelika.sawicka@awf.gda.pl (A.K.S.); 2 Mass Spectrometry Laboratory, Institute of Biochemistry and Biophysics, Polish Academy of Sciences, Pawinskiego 5a, 02-106 Warsaw, Poland; emi.sambor@gmail.com

**Keywords:** cardiovascular disease, endothelial dysfunction, atherosclerosis, cholesterol, leukocytes

## Abstract

L-carnitine supplementation elevates plasma trimethylamine-N-oxide (TMAO), which may participate in atherosclerosis development by affecting cholesterol metabolism. The aim of the current study was to determine the effect of increased plasma TMAO on biochemical markers in the blood following cessation of L-carnitine supplementation. The follow-up measurements were performed on subjects who completed 24 weeks of L-carnitine or placebo supplementation protocol. Blood samples were taken after finishing the supplementation and then 4 and 12 months following the supplementation withdrawal. Four months after cessation of L-carnitine supplementation, plasma TMAO concentration reached a normal level which was stable for the following eight months. During this period, no modifications in serum lipid profile and circulating leukocyte count were noted. TMAO implications in health and disease is widely discussed. The results of this study demonstrate no adverse effects of elevated plasma TMAO, induced by L-carnitine, on the measured parameters at 4 and 12 months after withdrawal of supplementation.

## 1. Introduction

Atherosclerosis is a leading cause of vascular disease worldwide, even if several major modifiable risk factors have been identified [1]. The early atherosclerotic lesion is characterized by the accumulation of arterial foam cells derived mainly from cholesterol-loaded macrophages [2]. Thus, cholesterol metabolism has generated considerable notoriety for its causative role in atherosclerosis [3]. Recent studies suggest that trimethylamine N-oxide (TMAO) may participate in the development of atherosclerosis [4]. By reducing reverse cholesterol transport, TMAO elevates cholesterol uptake in the vascular wall, leading to macrophage foam cell formation and atherosclerotic lesion development [4,5].

TMAO may be produced by the intestinal microbiota using L-carnitine as a substrate [5]. Therefore, L-carnitine has been suggested as a potential link between red meat consumption and atherosclerosis development [5]. L-carnitine is consumed not only from red meat but as a supplement due to its potential “fat burning” properties [6]. In fact, dietary L-carnitine supplementation induces TMAO elevation in human blood [7,8,9,10].

The aim of the current study was to determine the effect of increased plasma TMAO on biochemical markers in the blood following cessation of L-carnitine supplementation.

## 2. Materials and Methods

### 2.1. Subjects

The follow-up measurements were performed on subjects who had completed a study evaluating the effect of L-carnitine supplementation on skeletal muscle function [11]. Due to personal reasons, follow-up measurements were discontinued by two subjects. Therefore, the results of 18 women in the age range of 65–70 years, L-carnitine (*n* = 10) and placebo (*n* = 8), were used for statistical analyses.

### 2.2. Study Procedure

**Figure 1:**
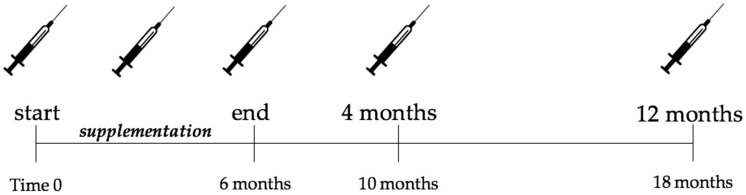
Study time line. Syringes indicate blood sampling. The results of the supplementation protocol have been presented elsewhere [10,11].

Subjects visited the laboratory after 24 weeks supplementation of either 1500 mg L-carnitine-L-tartrate or isonitrogenous placebo per day, as described previously [11], and 4 and 12 months following the cessation of supplementation (Figure 1). The study protocol was approved by the Independent Bioethics Commission for Research at Medical University of Gdansk (NKBBN/354-304/2015 and NKBBN/354-201/2017). All subjects gave written informed consent for participation in the study.

### 2.3. Fish Consumption Habits

Nutritional intake patterns, especially fish and seafood, have a great impact on TMAO production in the human body [12]. Therefore, to assess the frequency of fish consumption, a specific survey was constructed in a comprehensible form that was easily filled out by all subjects. The survey used quantitative research methods to identify “frequency consumption groups” [13]:

F0—never/occasionally,

F1—1–2 times per month,

F2—once per week,

F3—2–5 times per week,

F4—every day.

### 2.4. Blood Sampling

Fasting blood samples were taken from the antecubital vein. White blood cell (WBC) count and differential leukocyte count were determined using an automated hematology analyzer (Sysmex XT 2000, Global Medical Instrumentation, Inc (Mundelein, IL, USA)) in the whole blood. Serum and EDTA-plasma were obtained by centrifugation at 2000× *g* at 4 °C for 10 min. Samples were stored at −80 °C until analyses.

### 2.5. Biochemical Determination

Plasma TMAO was determined by the UPLC/MS/MS method as described previously [14]. Total cholesterol (TCh), high-density lipoprotein (HDL) cholesterol, low-density lipoprotein (LDL) cholesterol, and triglycerides (TG) were determined using Cobas6000 (Roche Diagnostics, Mannheim, Germany).

### 2.6. Statistical Analyses

Statistical calculations were performed using Statistica 13.1 (Dell Inc., Tulsa, OK, USA). The analysis of variance (ANOVA) for repeated measurements was performed to examine the interaction between the treatment and time, with Tukey–Kramer post hoc comparisons. A probability level of *p* < 0.05 was considered statistically significant. All data are expressed as mean ± standard error (SE).

## 3. Results

**Figure 2:**
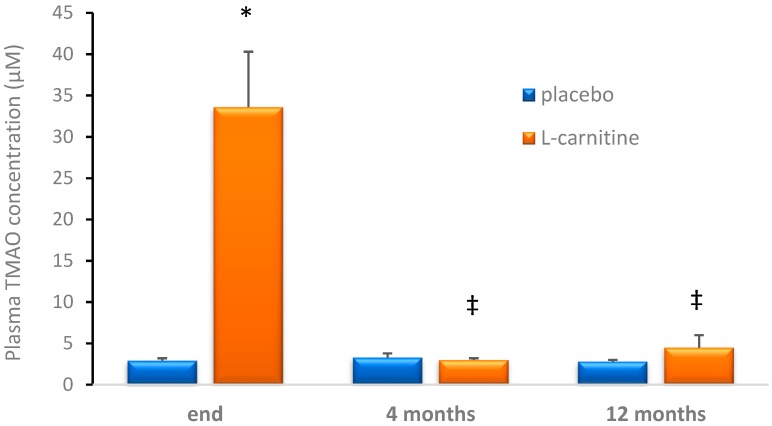
Plasma trimethylamine-N-oxide (TMAO) concentrations in placebo and L-carnitine groups. * *p* < 0.001 as compared to placebo group at the same time point, ‡ *p* < 0.001 as compared to the end of supplementation in the same group.

At the end of the supplementation protocol, the plasma TMAO concentration reached 33.6 ± 6.7 μM in the L-carnitine group, whereas it was 2.9 ± 0.3 μM in the placebo group (Figure 1). Four months after cessation of supplementation, plasma TMAO decreased to a level comparable to the placebo group and remained stable for the following eight months (Figure 2).

During the same time intervals, circulating lipid profile metabolites were not modified by either the supplementation or the washout period (Table 1).

Similarly, the leukocyte count remained unchanged and was not different between the groups (Table 2).

No differences in fish consumption habits were noted (Table 3).

## 4. Discussion

The main finding of the current study is that four months after cessation of L-carnitine supplementation, plasma TMAO concentration reached a normal level which was stable for the following eight months. During this period, no modifications in serum lipid profile and circulating leukocyte count were noted.

Elevated plasma TMAO has been suggested as a predictor of poor prognosis in cardiovascular disease patients associated with increased risk of major adverse cardiovascular events or death [15,16,17,18]. However, adjustment for kidney function abolishes the statistical significance of plasma TMAO in relation to patients’ mortality [18]. It is worth noting that cardiovascular disease and kidney disease are closely interrelated [19] and diminished renal function is strongly associated with morbidity and mortality in heart failure patients [20]. Moreover, at the beginning of this century, elevated plasma TMAO levels were detected in patients with chronic renal disease [21] and were suggested as a marker of ischemic kidney damage [22]. Glomerular filtration rates of the subjects participating in this study were within the normal range [10], and four months after cessation of L-carnitine treatment, TMAO decreased to a level comparable to the values observed before supplementation started [10].

Higher risks of all-cause and cardiovascular disease mortality have been linked to red meat consumption [23], with TMAO as a potential link between red meat consumption and atherosclerosis development [5]. However, in the German adult population, meat consumption was not related to plasma TMAO [24]. Moreover, a minor increase in plasma TMAO was observed following red meat and processed meat consumption, whereas fish induced significant elevation of plasma TMAO [25]. Furthermore, Veeravalli et al. [26] reported that TMAO does not increase plasma cholesterol or act as a proatherogenic molecule under normal dietary conditions. Levels of red meat and fish consumption of the subjects participating in this study were similar in both groups [11] and did not differ from nutritional habits in this region [13].

TMAO is produced using L-carnitine as a substrate [5]; thus, L-carnitine supplementation elevates plasma TMAO [7,8,9,10]. In patients with inborn errors of metabolism who require L-carnitine supplementation, the average plasma TMAO reached 120 µM, which was ~45-fold higher compared with patients without a diagnosed genetic disorder on a standard omnivorous Western diet that was not supplemented by L-carnitine [8]. Vallance et al. [9] reported that L-carnitine treatment (1000 mg daily for >1 year) of patients with mitochondrial disorders induced a ~12-fold increase in median TMAO concentration (3.54 vs. 43.26 μM). It is worth noting that one subject with chronic kidney disease had a marked elevation of plasma TMAO at baseline (33.98 μM), which increased 3-fold on oral L-carnitine therapy (33.98 vs. 101.6 μM) [9]. Healthy aged women, participating in the current study, were supplemented by 1500 mg L-carnitine-L-tartrate per day for 24 weeks [11]. A 10-fold increase of plasma TMAO was noted after 12 weeks of supplementation and remained elevated for the next 12 weeks [10]. Considering the increase of relative risk for all-cause mortality by 7.6% per each 10 μM increment of TMAO [27], and TMAO elevation due to L-carnitine supplementation [7,8,9,10], the negative impact of L-carnitine should have been observed. However, L-carnitine has been used as a treatment for cardiovascular disease patients for decades with no detrimental effects reported (for review, see [28,29,30]). Even if the positive effect of L-carnitine has been recently negated, as there is no significant marginal benefit in terms of all-cause mortality, heart failure, unstable angina, or myocardial reinfarction [28], it has still not been shown to be a harmful compound—although much higher doses (e.g., 6 g/day for a period of 1 year) have been tested in patients with acute anterior myocardial infarction [31]. Furthermore, genetic variants of organic cation transporter 2 reduce cardiac uptake of carnitine, leading to heart failure [32].

The number of subjects examined in our study was very small. Another limitation is the relatively short period of supplementation. Since atherosclerosis develops over the course of years, 24 weeks may be not sufficient to induce any changes in the determined metabolites. However, we have noted that a 10-fold plasma TMAO increase, induced by L-carnitine supplementation, does not affect inflammatory markers (i.e., vascular cell adhesion molecule, intercellular adhesion molecule, L-selectin, P-selectin, C-reactive protein, tumor necrosis factor α, and interleukin-6 [10]) and oxidative stress markers (unpublished). Moreover, the neutrophil-to-lymphocyte ratio (NLR), an index of systemic inflammation associated with subclinical atherosclerosis [33], during and after the supplementation remained at a level ≤ 1.8, which was comparable to the control subjects [34]. Nevertheless, it remains unknown whether other inflammatory markers, not determined in previous studies, are influenced by carnitine supplementation.

## 5. Conclusions

TMAO implications in health and disease are widely discussed [35,36,37], but it is still debated whether it is a mediator in human disease development or a causative agent in the disease process [38,39].

## Figures and Tables

**Table 1:** Serum lipid metabolites in L-carnitine and placebo groups after 24 weeks of supplementation (end) and 4 and 12 months after cessation of supplementation.

	L-carnitine			Placebo		
	End	4 Months	12 Months	End	4 Months	12 Months
TCh (mg·dL^−1^)	217 ± 12	213 ± 16	211 ± 13	199 ± 15	211 ± 10	202 ± 11
HDL (mg·dL^−1^)	67 ± 5	72 ± 6	70 ± 5	61 ± 4	72 ± 7	67 ± 5
LDL (mg·dL^−1^)	127 ± 10	119 ± 14	115 ± 12	117 ± 13	120 ± 10	117 ± 7
TG (mg·dL^−1^)	114 ± 18	116 ± 14	130 ± 22	103 ± 18	98 ± 12	89 ± 14

TCh: total cholesterol; HDL: high-density lipoprotein cholesterol; LDL: low-density lipoprotein cholesterol; TG: triglycerides.

**Table 2:** Circulating white blood cell counts in L-carnitine and placebo groups after 24 weeks of supplementation (end) and 4 and 12 months after cessation of supplementation.

	L-carnitine			Placebo		
	End	4 Months	12 Months	End	4 Months	12 Months
Leuko (10^9^·L^−1^)	5.7 ± 0.5	6.1 ± 0.6	6.6 ± 0.8	5.4 ± 0.4	5.7 ± 0.5	5.5 ± 0.4
Neutro (10^9^·L^−1^)	3.0 ± 0.3	3.4 ± 0.5	3.7 ± 0.5	3.0 ± 0.2	3.0 ± 0.4	2.7 ± 0.2
Lympho (10^9^·L^−1^)	2.0 ± 0.2	2.0 ± 0.2	2.1 ± 0.2	1.8 ± 0.2	2.1 ± 0.2	2.1 ± 0.2
NLR	1.6 ± 0.2	1.7 ± 0.2	1.8 ± 0.2	1.7 ± 0.2	1.5 ± 0.2	1.4 ± 0.1
Mono (10^9^·L^−1^)	0.51 ± 0.04	0.51 ± 0.03	0.54 ± 0.05	0.45 ± 0.06	0.46 ± 0.06	0.45 ± 0.04
Platelets (10^9^·L^−1^)	279 ± 16	272 ± 12	291 ± 13	249 ± 19	248 ± 18	253 ± 23

Leuko: leukocytes; Neutro: neutrophils; Lympho: lymphocytes; NLR: neutrophil-to-lymphocyte ratio; Mono: monocytes.

**Table 3:** The frequency of fish consumption.

	L-carnitine		Placebo	
	Median	Range	Median	Range
Cod	F1	F0–F3	F1	F0–F3
Salmon	F1	F0–F2	F2	F0–F3
Mackerel	F1	F0–F2	F1	F0–F2
Herring	F1	F0–F3	F1	F0–F3
Trout	F0	F0–F1	F1	F0–F3
Tuna	F0	F0–F1	F0	F0–F2
Flounder	F0	F0–F1	F0	F0–F1
Hake	F0	F0–F1	F0	F0–F1
Eel	F0	F0–F1	F0	F0–F1
Pollock	F0	F0–F1	F0	F0–F1

F0: never/occasionally; F1: 1–2 times per month; F2: once per week; F3: 2–5 times per week.
